# Progress of the National Insurance Bill

**Published:** 1911-09-23

**Authors:** 


### PROGRESS OF THE NATIONAL INSURANCE BILL.

### FRIENDLY SOCIETIES AND THE BILL.

The Chancellor of the Exchequer has consented to receive a deputation consisting of representatives of the British Medical Alliance and the National Conference of Friendly Societies to discuss clauses 13 and 14 of the National Insurance Bill. It will be remembered that in the original Bill clause 13 provided that the medical benefits should be administered by approved friendly societies and local health committees, but that during its progress through the Committee of the House of Commons an amendment proposed by Dr. Addison, which provided that the control of the medical service under the Bill should be in the hands of the health committees and not under the control of friendly societies, was carried by an overwhelming majority. It appears to be the desire of the organisations to be represented by the deputation to reopen the whole question, for at a special conference of the Medical Alliance (which represents nearly eighty friendly societies' medical associations), held at Luton on September 16, a protest was entered against the action of the House of Commons in destroying the powers of friendly societies to make their own arrangements for medical attendance under the National Insurance Bill. It is interesting to recall that Dr. Addison's amendment was carried by a majority of 372.

